# Supplementary material for: Long-term isolation of European steppe outposts boosts the biome’s conservation value
Source: Nat Commun. 2020 Apr 23;11:1968. doi: 10.1038/s41467-020-15620-2 (PMC7181837; doi:10.1038/s41467-020-15620-2)
Supplement: Supplementary file 1 — Supplementary Information [file 41467_2020_15620_MOESM1_ESM.docx]

1. Long-term isolation of European steppe outposts boosts the biome’s conservation value

Kirschner et al.

*Correspondence*: Philipp Kirschner, [philipp.kirschner@gmail.com](mailto:philipp.kirschner@gmail.com),

**Supplementary Information**

# Supplementary Methods

### Restriction site associated DNA sequencing

- - 1. The amount of input DNA was adapted species-specifically. Briefly, 150 ng DNA from plants, 40-100 ng DNA of the ant *Plagiolepis taurica,* and 250 ng DNA from the grasshoppers *Omocestus petraeus* and *Stenobothrus nigromaculatus* were used per individual for restriction digestion with the enzymes PstI (plant taxa and *P. taurica*) or SbfI (*O. petraeus* and *S. nigromaculatus*). A double barcoding approach was used to decrease the number of adapters necessary to pool 84–96 individuals into a single library. A six-base-pair (bp) P2 barcode and a 14-bp P1 barcode that differed by at least three bases from each other were selected to avoid erroneous assignment of fragments due to sequencing problems. The P1 adapter was ligated to the restricted samples overnight at 16 °C. After having been barcoded with different P1 barcodes, samples were sheared in a two-minute, focused ultrasonication program in a Covaris sonicator (M220 series, Covaris Inc., Woburn, USA) to obtain average fragment lengths of 400 bp. To remove undesired fragment lengths from each pool, left- and right-side size selection steps were done, using ×0.7 and ×0.55 volume of SPRIselect reagent (Beckman Coulter, California, USA). After ligation of P2 adaptors, DNA content of each sample was quantified, and samples were pooled to be equally represented in the final sample. Further, size selection steps were done on the left side with ×0.55 volume of SPRI reagent before and after the 18 cycles of PCR amplification in Phusion Master Mix (Thermo Fisher Scientific, Waltham, USA). The libraries were sequenced on a HiSeq2000 sequencer (Illumina) at CSF Vienna (http://csf.ac.at/facilities/next-generation-sequencing/) as 100-bp single reads.

### Climatic data for ecological niche modelling (ENM)

Worldclim^1^ data have low precision for precipitation values in mountainous areas such as the Alps, leading to a potentially poor representation of the precipitation regime of inner-Alpine dry valleys^1^⁠. As such, the Wordclim precipitation variables might distort the niche of inner alpine dry-valley dwelling biota in the framework of ecological niche modelling. To account for this effect, a correction of five Worldclim^1^ bioclim precipitation variables for the area of the dry valleys was done, utilizing the observational data of climate stations in the vicinity of these valleys. In detail, precipitation records for 1960-1990 were obtained from 23 climate stations from the *Zentralanstalt für Meteorologie und Geodynamik* (ZAMG; [www.zamg.ac.at](http://www.zamg.ac.at/)), *Federal Office of Meteorology and Climatology* (MeteoSwiss; [http://www.meteosuisse.admin.ch](http://www.meteosuisse.admin.ch/)), and Météo-France ([http://www.meteofrance.com](http://www.meteofrance.com/)) (Supplementary Figure 6). From the Embrun station in the French Alps, only monthly mean precipitation values of 1981–2010 were available. Based on mean monthly precipitation records of the available periods, values for *Annual Precipitation* (bio12), *Precipitation of Wettest Month* (bio13), *Precipitation of Driest Month* (bio14), *Precipitation of Wettest Quarter* (bio16) and *Precipitation of Driest Quarter* (bio17) were re-calculated at the locations of the climate stations^2^⁠. For each of the five precipitation variables (bio12, bio13, bio14, bio16, bio17), differences between the values derived from the climate stations, and those of the Worldclim^1^ variables at the same locations, were calculated (Supplementary Table 8). These differences were used to interpolate surfaces of deviations using empirical Bayesian kriging (see Supplementary Figure 6), a probabilistic interpolation method that accounts for the error in estimating the underlying semivariogram (= function of distance and direction separating two locations) through repeated simulations^3^⁠. This step was performed separately for eastern and western valleys, using eastern and western climate stations, respectively, to avoid interpolation over large and heterogeneous areas (Supplementary Table 8). Finally, the resulting surfaces were added to each of the five original bioclim variables for the area encompassing the inner-Alpine dry valleys (while leaving the rest of the layer unaltered). These dry valley-specific variables were used along with the unaltered temperature related bioclim variables (bio1 – bio11) and the unmodified *Precipitation Seasonality* (bio15) for all subsequent analyses. Bio18 and bio19 could not be corrected using the above approach and were therefore excluded as predictors in subsequent analyses.

### ENM of present and Last Glacial Maximum (LGM) distributions

Species-specific fine tuning of models was performed to improve model performance and transferability by avoiding overfitting and ensuring sufficient discriminatory ability ^4–7^⁠. In detail, combinations of regularization parameters (β= 0.5; 1; 1.5; 2; 2.5; 3) and feature classes (L: linear; H: hinge; LQ: linear-quadratic; and LQH: linear-quadratic-hinge) have been evaluated in regard to model performance using the R package ENMeval version 0.2.2^8^⁠ under a jackknife (k=n) cross validation replication regime. To increase the transferability of the present-day niche models to paleo-climatic conditions, the contribution and permutation importance of each of the eleven uncorrelated bioclim variables was assessed using jackknife tests^9^⁠. Variables that caused a pronounced increase in test gain or area under the curve (AUC) when omitted (here limited to >10%), or with percentage contribution and permutation importance lower than 1%, were removed prior to paleo-projection^10,11^⁠. Consequently, bio10 was removed (*Euphorbia seguieriana, O. petraeus* and *P. taurica*), as well as bio11 (*E. seguieriana*) and bio12 (*O. petraeus*).

### ENM and background test of niche divergence

Extrazonal and zonal sampling localities of steppe species differ in their macro-climatic conditions. This was shown by using extrazonal and zonal occurrences of the six steppe species and by applying the Background Test as described by McCormack et al.^12^. This multivariate method does not rely on ENMs but rather compares differences in the environmental background to determine if two sets of localities are more or less similar than expected based on their environmental background^12,13–^. For each species, climatic values of the same bioclim variables as used for ENM were extracted at the species localities as were 1000 random background points using the functionalities of ArcGIS v. 10. 4 (ESRI, Redland, CA). Background area was defined as circular buffer of 100 km in diameter around the occurrence points. PCA were performed and for each species, the first four component scores (PC1–PC4) and the differences for each PC axis between zonal and extrazonal localities were calculated and compared with a null distribution^12,13^ (generated by calculating the difference between background points using a bootstrapping approach and 1000 resamples). The null hypothesis that two sets of occurrences are as similar as expected based on their environmental background is rejected if the observed difference in PC score is lower (= niche conservatism) or higher (= niche divergence) than the 95% confidence limits of the null distribution. The tests were performed using the R package *boot*^14^ *(*version 1.3-23) following the script provided by Johnson et al.^13^.

### Delimitation of the extent of the extrazonal steppes

Steppes in central and Eastern Europe were delimited based on Niklfeld^15^, whereas all subcategories of the Forest-steppe zone were summarized into a single layer. Steppes North of the Alps and on the Italian peninsula were delimited based on the distribution of *Stipa capillata* Meusel et al.^16⁠^. The steppes in the inner-Alpine dry valleys are defined as in Braun-Blanquet^17⁠^. Habitat classification data of the European Environment Agency (http://natura2000.eea.europa.eu/) were utilized to outline steppe patches in Southern France (i.e. Lower Durance Valley, Massif Central, Montagne de la Moure, Caussols, Causse Mejan, Crau, Ardeche Valley). On the Iberian Peninsula, steppe delimitation is based on the distribution of *Ononis tridentata*, a characteristic plant of the Iberian edaphoxerophilous steppes^18^⁠. All these data were finally summarized in a map of the extrazonal steppes in Europe (Figure 1A). Formation of extrazonal steppes by definition depends on certain local climatic, topographic, and/or edaphic conditions (see Introduction). Therefore, the map depicts areas where the potential natural vegetation comprises steppes. These areas, however, are never exclusively covered by steppes and must certainly not be interpreted in this way.

# Supplementary Note

### Niche divergence

For each species, the first four component scores (PC1–PC4) explained 90–92% of the total variation (Supplementary Table 5). Evidence of niche divergence between zonal and extrazonal localities was detected in all species and in 14 out of 24 niche axes tested. For each species, significant divergence was detected in two to three PC axes explaining a cumulative variance of 21–55 % (Supplementary Table 5). In summary, extrazonal populations occupy areas that are climatically significantly more divergent from those of zonal populations than expected by chance.

Supplementary Table 1. Summary of Restriction site associated DNA Sequencing data specifications (raw and analysis-specific); settings for SNP calling in Stacks^19^⁠. Abbreviations in species column, AO: *Astragalus onobrychis*, ES: *Euphorbia seguieriana*, SC: *Stipa capillata*, OmP: *Omocestus petraeus*, PT: *Plagiolepis taurica*, SN: *Stenobothrus nigromaculatus*.

| Species | Median # of reads per sample | Median coverage | STACKS denovo_map^19^⁠ settings | # of loci (=SNPs) for Bayesian clustering | % of missing data per locus allowed for in Bayesian clustering | # of loci/ # of SNPs & invariant sites in RaxML datasets | % of missing data per locus allowed for in RaxML |
| --- | --- | --- | --- | --- | --- | --- | --- |
| AO | 1171508 | 19 | -m 10, -n 3, -M 3 | 6475 | 45 | 5305/12433 | 75% |
| ES | 722785 | 17 | -m 7, -n 5, -M 5 | 4930 | 35 | 4802/11560 | 75% |
| SC | 1119575 | 8 | -m 5, -n 5, -M 5 | 6201 | 45 | 5993/10536 | 75% |
| OmP | 224937 | 20 | -m 5, -n 2, -M 2 | 13037 | 84 | 5778/21124 | 85% |
| PT | 699360 | 18 | -m 7, -n 2, -M 2 | 6710 | 46 | 3889/13330 | 75% |
| SN | 249215 | 14 | -m 5, -n 2, -M 2 | 3899 | 73 | 8223/10432 | 75% |

Supplementary Table 2: Summary of primers and PCR conditions used for amplification of mitochondrial Cytochrome Oxidase Subunit 1 gene from *Omocestus petraeus* and *Plagiolepis taurica*, length of sequenced fragment, and number of sequenced individuals and populations.

| **Target** | **Primer** | | **PCR conditions** | **Length of amplified fragment in base pairs after trimming** | **Primer reference** | **Sequenced Individuals / populations** |
| --- | --- | --- | --- | --- | --- | --- |
| cDNA template | Forward | 5’-GGTCAACAAATCATAAAGATATTGG-3’ | 94 °C – 6’; 30 cycles : 94 °C – 1’, 58 °C – 30’’, 72 °C; 72 °C - 10’ | 660 (*O*. *petraeus*) | LCO1490^20^⁠ |  |
|  | Reverse | 5’-AAAAATGTTGAGGGAAAAATGTTA-3’ |  |  | UEA8^21^⁠ |  |
| *Omocestus* *petraeus* DNA extracts | Forward | 5’-CTCTAATTGGAGATGATCAAATC-3’ | 94 °C – 6’; 30 cycles : 94 °C – 1’, 58 °C – 30’’, 72 °C; 72 °C - 10’ | 642 | OpetF3  (This study) | 159 / 54 |
|  | Reverse | 5’-ATCTATGCTGCAGGGGATT-3’ |  |  | OpetR1  (This study) |  |
| *Plagiolepis* *taurica* DNA extracts | Forward | 5’-GGTCAACAAATCATAAAGATATTGG-3’ | 94 °C – 6’; 30 cycles : 94 °C – 1’, 58 °C – 30’’, 72 °C; 72 °C - 10’ | 614 | LCO1490^20⁠^ | 148 / 58 |
|  | Reverse | 5’-AAAAATGTTGAGGGAAAAATGTTA-3’ |  |  | UEA8^21^⁠ |  |

Supplementary Table 3. Summary statistics for the species-specific model-tuning for ecological niche modelling of six steppe dwelling species. Optimal settings (combining optimal feature class for variable transformation and regularization parameter β) that minimize overfitting and then maximize discriminatory ability has been selected based on OR10, 10% training omission rate; and ORMTP, omission rate based on the minimum training presence threshold as well as AUCTEST, mean area under the curve of the receiver-operating characteristic plot on the testing data. Number of localities (n) used for modelling after removing occurrence records within a spherical distance of 5 km.

| Species | n | Feature | β | OR10 | ORMTP | AUCTest |
| --- | --- | --- | --- | --- | --- | --- |
| *Astragalus onobrychis* | 85 | LQH | 3 | 0.107 | 0.012 | 0.814 |
| *Euphorbia seguieriana* | 50 | L | 1.5 | 0.122 | 0.041 | 0.746 |
| *Omocestus petraeus* | 46 | LQ | 0.5 | 0.156 | 0.022 | 0.794 |
| *Plagiolepis taurica* | 54 | LQ | 2.5 | 0.113 | 0.019 | 0.754 |
| *Stenobothrus nigromaculatus* | 41 | H | 2.5 | 0.1 | 0.025 | 0.831 |
| *Stipa capillata* | 104 | H | 3 | 0.107 | 0.01 | 0.788 |

Supplementary Table 4. Divergence on niche axes between zonal and extrazonal localities of the six steppe species. Bold values indicate significant niche divergence (D) or conservatism (C) compared with null distribution (in parentheses) based on background divergence between the respective geographic ranges.

| Pairwise comparison  (zonal vs. extrazonal) | Niche axes | | | |
| --- | --- | --- | --- | --- |
|  | PC1 | PC2 | PC3 | PC4 |
| *Astragalus onobrychis* | 2.63 | **1.47** | **0.99** | **0.90** |
| Null distribution (95% CI) | (2.45, 2.73) | (1.13, 1.41) | (0.22, 0.45) | (0.23, 0.37) |
| Result | NS | **D** | **D** | **D** |
| % variance explained | 43 | 25 | 15 | 6 |
| *Euphorbia seguieriana* | **1.76** | **1.57** | **1.63** | **0.76** |
| Null distribution (95% CI) | (2.07, 2.40) | (1.71, 1.95) | (0.61, 0.84) | (0.42, 0.55) |
| Result | **C** | **C** | **D** | **D** |
| % variance explained | 44 | 26 | 16 | 5 |
| *Stipa capillata* | **2.12** | **0.92** | **1.63** | **0.84** |
| Null distribution (95% CI) | (2.37, 2.68) | (1.11, 1.41) | (0.36, 0.59) | (0.43, 0.57) |
| Result | **C** | **C** | **D** | **D** |
| % variance explained | 40 | 29 | 15 | 6 |
| *Omocestus petraeus* | 2.87 | **1.14** | **1.52** | **0.54** |
| Null distribution (95% CI) | (2.64, 2.94) | (1.19, 1.47) | (0.54, 0.74) | (-0.02, 0.11) |
| Result | NS | **C** | **D** | **D** |
| % variance explained | 44 | 26 | 13 | 7 |
| *Plagiolepis taurica* | 2.80 | **1.40** | **0.97** | **1.14** |
| Null distribution (95% CI) | (2.63, 2.91) | (1.07, 1.36) | (0.37, 0.58) | (0.49, 0.64) |
| Result | NS | **D** | **D** | **D** |
| % variance explained | 42 | 27 | 13 | 7 |
| *Stenobothrus nigromaculatus* | 3.70 | **1.20** | **0.56** | **0.84** |
| Null distribution (95% CI) | (2.86, 3.15) | (1.53, 1.78) | (0.63, 0.82) | (0.36, 0.49) |
| Result | **D** | **C** | **C** | **D** |
| % variance explained | 49 | 24 | 13 | 5 |

Supplementary Table 5. Summary of phylogenetic diversity complementarity calculations. Values are shown for all species and were seperately calculated for all extrazonal steppes and all zonal steppes.

|  | *Astragalus onobrychis* | *Euphorbia seguieriana* | *Stipa  capillata* | *Omocestus petraeus* | *Plagiolepis taurica* | *Stenobothrus nigromaculatus* |
| --- | --- | --- | --- | --- | --- | --- |
| Extrazonal steppes | 0.0549 | 0.0605 | 1.7382 | 0.0616 | 0.0187 | 0.0243 |
| Zonal  steppes | 0.0094 | 0.0257 | 0.6181 | 0.0075 | 0.0052 | 0.0020 |

Supplementary Table 6. Number of populations and, in brackets, individuals included in respective analyses. Abbreviations in species column, AO: *Astragalus onobrychis*, ES: *Euphorbia seguieriana*, SC: *Stipa capillata*, OmP: *Omocestus petraeus*, PT: *Plagiolepis taurica*, SN: *Stenobothrus nigromaculatus*.

| **Species** | Ecological niche modelling | Bayesian Clustering | RAxML trees as shown in Figure 3 | RAxML trees for  PD/ PE calculations |
| --- | --- | --- | --- | --- |
| AO | 85 | 70 (199) | 70 (70) | 70 (175) |
| ES | 50 | 55 (160) | 55 (55) | 55 (153) |
| SC | 104 | 92 (262) | 92 (92) | 92 (235) |
| OmP | 46 | 55 (160) | 55 (55) | 55 (123) |
| SN | 41 | 38 (110) | 38 (38) | 38 (70) |
| PT | 54 | 60 (145) | 60 (60) | 60 (123) |
| TOTAL | 380 | 370 (1036) | 370 (370) | 370 (879) |

Supplementary Table 7: Details on the species used as outgroup taxa for phylogenetic tree construction, including collection details and Short Read Archive (SRA) accession numbers.

| **Taxon** | **No of ind per pop (RADseq)** | **SRA accession number** | **Country** | **Province** | **Region** | **Locality** | **Collector** | **Latitude** | **Longitude** | **Altitude** |
| --- | --- | --- | --- | --- | --- | --- | --- | --- | --- | --- |
| **Plants** |  |  |  |  |  |  |  |  |  |  |
| *Astragalus goktschaicus* | 1 | SAMN 10612750 | Armenia | Gegharkunik | Tsovagyugh | Gas station on M4 ca. 10 km N Sevan | M. Falch  B. Frajman | 40.63916 | 44.94527 | 1950 |
| *Astragalus goktschaicus* | 1 | SAMN 10612772 | Turkey | Northeast Anatolia | Ardahan | Göle, Roadside on D060 about 3 km SW Göle (GPS-32) | B. Frajman Ch. Gilli M. Falch P. Schönswetter | 40.77222 | 42.58611 | 2060 |
| *Astragalus goktschaicus* | 1 | SAMN 10612783 | Turkey | Erzurum | Erzurum | Ilıca, Roadside on D925 between Erzurum and Pazaryolu; about 2.9 km E Rizekent Köyü (GPS-87) | B. Frajman Ch. Gilli M. Falch P. Schönswetter | 40.15888 | 41.02416 | 2050 |
| *Stipa lagascae* | 1 | SAMN14246446 | Spain | Castilla y León | Segovia | Cuellar, hills N of SE entrance of town | A. Hilpold | 41.39055 | -3.70194 | 840 |
| *Stipa lagascae* | 1 | SAMN14246447 | Spain | Cataluña | Lleida | 0.5 km N of Algerri, 0.3 km NE of castle | A. Hilpold  M. Prahmsohler | 41.81972 | 0.64027 | 360 |
| *Euphorbia niciciana* | 1 | SAMN10316023 | Greece | Epirus |  | Western Macedonia, Epirus, Road between Konitsa and Elefthero, ca. 7.5 km E of Konitsa | B. Frajman  P. Schönswetter | 40.06777 | 20.83305 | 893 |
| *Euphorbia niciciana* | 1 | SAMN10316021 | Serbia | Šumadija |  | E of Ovčar Banja, between čačak and Užice | B. Frajman  P. Schönswetter | 44.44111 | 21.95583 | 317 |
| *Euphorbia niciciana* | 1 | SAMN10316031 | Turkey | Bartin |  | Kurucasile, roadside between Karaman and Curunlu | M. Falch | 41.82194 | 32.65833 | 223 |
| **Animals** |  |  |  |  |  |  |  |  |  |  |
| *Plagiolepis pygmaea* | 1 | SAMN14167108 | Croatia | Primorje-Gorski Kotar | Rab | Path from top of Kamenjak to Mundanije, 0.5 km W of summit of Kamenjak | P. Kirschner | 44.77297 | 14.78327 | 335 |
| *Omocestus haemorrhoidalis* | 1 | SAMN14167106 | Italy | Aosta | Aosta | S-slope, 1.5 km NW of Morgex | E. Trucchi | 45.76642 | 7.02429 | 1071 |
| *Omocestus haemorrhoidalis* | 1 | SAMN14167107 | Italy | Piemont | Val Susa | SW-slopes, 0.7 km NW of Puys | E. Trucchi | 45.05128 | 6.73311 | 1532 |
| *Stenobothrus lineatus* | 1 | SAMN14167109 | Italy | Lazio | Rieti | Pian de Valli, 0.9 km SE of Terminillo | P. Kirschner | 42.45087 | 12.98464 | 1710 |

Supplementary Table 8. Differences between observed values at climate station and extracted values from Worldclim^1^ of five precipitation related bioclim variables (30 arc-sec resolution).

| **Station** | **Longitude** | **Latitude** | **Observed Values at climate station** | | | | | **Extracted values from Wordclim** | | | | | | **Differences between observed and extracted values** | | | | |
| --- | --- | --- | --- | --- | --- | --- | --- | --- | --- | --- | --- | --- | --- | --- | --- | --- | --- | --- |
|  |  |  | bio 12 | bio13 | bio14 | bio16 | bio 17 | | bio12 | bio13 | bio14 | bio16 | Bio 17 | bio12 | bio13 | bio14 | Bio 16 | bio17 |
| *Eastern* |  |  |  |  |  |  |  | |  |  |  |  |  |  |  |  |  |  |
| Rovereto | 11.050 | 45.890 | 966 | 104 | 58 | 280 | 188 | | 890 | 96 | 45 | 266 | 149 | -76 | -8 | -13 | -14 | -39 |
| Bozen | 11.330 | 46.500 | 703 | 92 | 28 | 255 | 90 | | 777 | 97 | 31 | 268 | 103 | 74 | 5 | 3 | 13 | 13 |
| Brixen | 11.650 | 46.720 | 694 | 114 | 21 | 307 | 69 | | 834 | 109 | 35 | 317 | 112 | 140 | -5 | 14 | 10 | 43 |
| Marienberg | 10.490 | 46.740 | 671 | 86 | 34 | 237 | 108 | | 1020 | 131 | 52 | 369 | 168 | 349 | 45 | 18 | 132 | 60 |
| Nauders | 10.500 | 46.900 | 680 | 97 | 35 | 265 | 105 | | 779 | 112 | 36 | 301 | 111 | 99 | 15 | 1 | 36 | 6 |
| Innsbruck | 11.385 | 47.261 | 841 | 128 | 36 | 349 | 124 | | 897 | 128 | 42 | 354 | 138 | 56 | 0 | 6 | 5 | 14 |
| Kufstein | 12.163 | 47.575 | 1289 | 172 | 72 | 487 | 242 | | 969 | 131 | 47 | 381 | 148 | -320 | -41 | -25 | -106 | -94 |
| Landeck | 10.564 | 47.139 | 722 | 99 | 38 | 280 | 128 | | 915 | 115 | 48 | 320 | 151 | 193 | 16 | 10 | 40 | 23 |
| Torbole Riva | 10.880 | 45.880 | 945 | 95 | 53 | 270 | 171 | | 867 | 92 | 48 | 260 | 154 | -78 | -3 | -5 | -10 | -17 |
| Trento | 11.120 | 46.070 | 1000 | 111 | 53 | 290 | 176 | | 899 | 98 | 45 | 271 | 151 | -101 | -13 | -8 | -19 | -25 |
| Belluno | 12.245 | 46.164 | 1298 | 144 | 68 | 396 | 233 | | 1088 | 125 | 53 | 333 | 182 | -210 | -19 | -15 | -63 | -51 |
| *Western* |  |  |  |  |  |  |  | |  |  |  |  |  |  |  |  |  |  |
| Château dOex | 7.140 | 46.480 | 1374 | 148 | 99 | 425 | 300 | | 1142 | 114 | 78 | 314 | 255 | -232 | -34 | -21 | -111 | -45 |
| Sion | 7.330 | 46.218 | 598 | 60 | 35 | 173 | 123 | | 686 | 79 | 40 | 199 | 134 | 88 | 19 | 5 | 26 | 11 |
| Formazza Ponte | 8.440 | 46.380 | 1175 | 142 | 61 | 355 | 222 | | 1610 | 173 | 102 | 496 | 326 | 435 | 31 | 41 | 141 | 104 |
| Col du Grand St. Bernard | 7.170 | 45.868 | 2237 | 225 | 128 | 648 | 408 | | 1879 | 182 | 136 | 509 | 426 | -358 | -43 | 8 | -139 | 18 |
| Ivrea | 7.880 | 45.460 | 883 | 108 | 40 | 298 | 129 | | 851 | 102 | 41 | 275 | 140 | -32 | -6 | 1 | -23 | 11 |
| Ceresole Reale | 7.250 | 45.430 | 1151 | 153 | 56 | 376 | 186 | | 1165 | 117 | 84 | 325 | 261 | 14 | -36 | 28 | -51 | 75 |
| Balme | 7.210 | 45.310 | 1199 | 170 | 55 | 413 | 220 | | 1562 | 147 | 111 | 413 | 359 | 363 | -23 | 56 | 0 | 139 |
| Lemie | 7.280 | 45.230 | 1243 | 181 | 54 | 474 | 188 | | 938 | 103 | 58 | 281 | 184 | -305 | -78 | 4 | -193 | -4 |
| Bardonecchia | 6.700 | 45.080 | 744 | 90 | 40 | 222 | 138 | | 1034 | 100 | 70 | 278 | 234 | 290 | 10 | 30 | 56 | 96 |
| Cuneo | 7.540 | 44.400 | 906 | 111 | 40 | 302 | 171 | | 770 | 91 | 34 | 243 | 149 | -136 | -20 | -6 | -59 | -22 |
| Geneve Cointrin | 6.128 | 46.248 | 967 | 94 | 63 | 262 | 219 | | 954 | 97 | 67 | 256 | 220 | -13 | 3 | 4 | -6 | 1 |
| Embrun | 6.496 | 44.566 | 727 | 86 | 45 | 224 | 147 | | 767 | 81 | 47 | 234 | 157 | 40.5 | -4.8 | 1.9 | 10.2 | 9.9 |


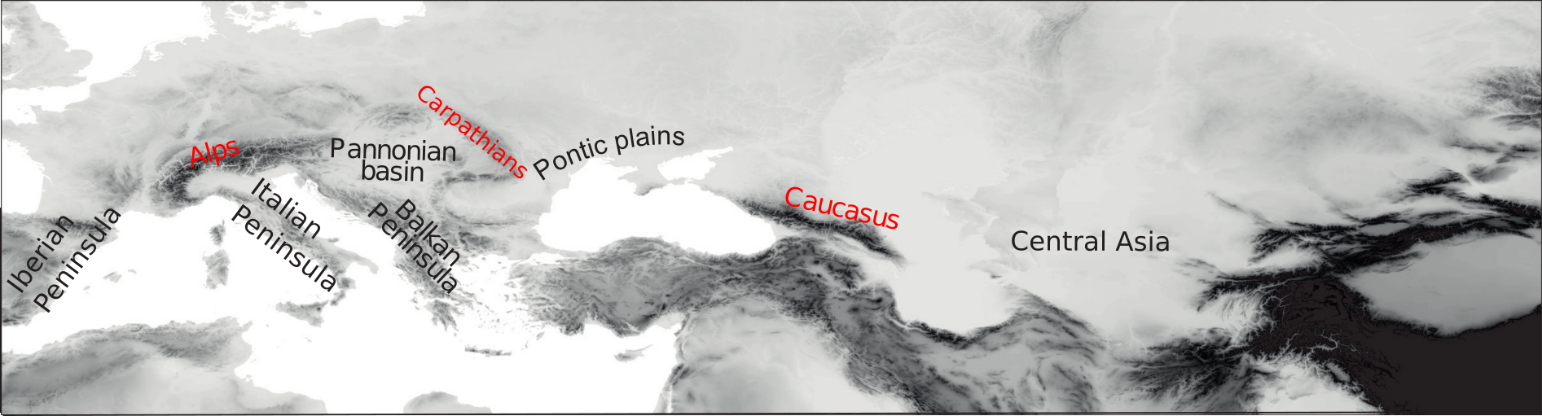


Supplementary Figure 1. Map depicting the toponyms used in the main text; names of mountain ranges are coloured in red.


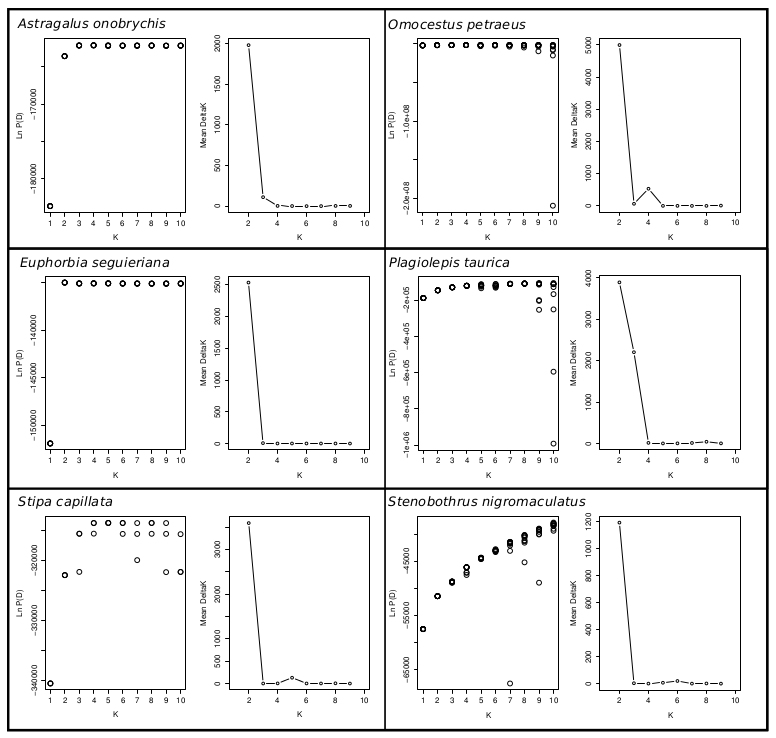


Supplementary Figure 2. Summary statistics of STRUCTURE^22^⁠ analyses of the six analysed species. Values of Ln probability of the model for each number of groups (K) are plotted against K values in the upper row and delta K value coefficients among runs against K values in the lower row.


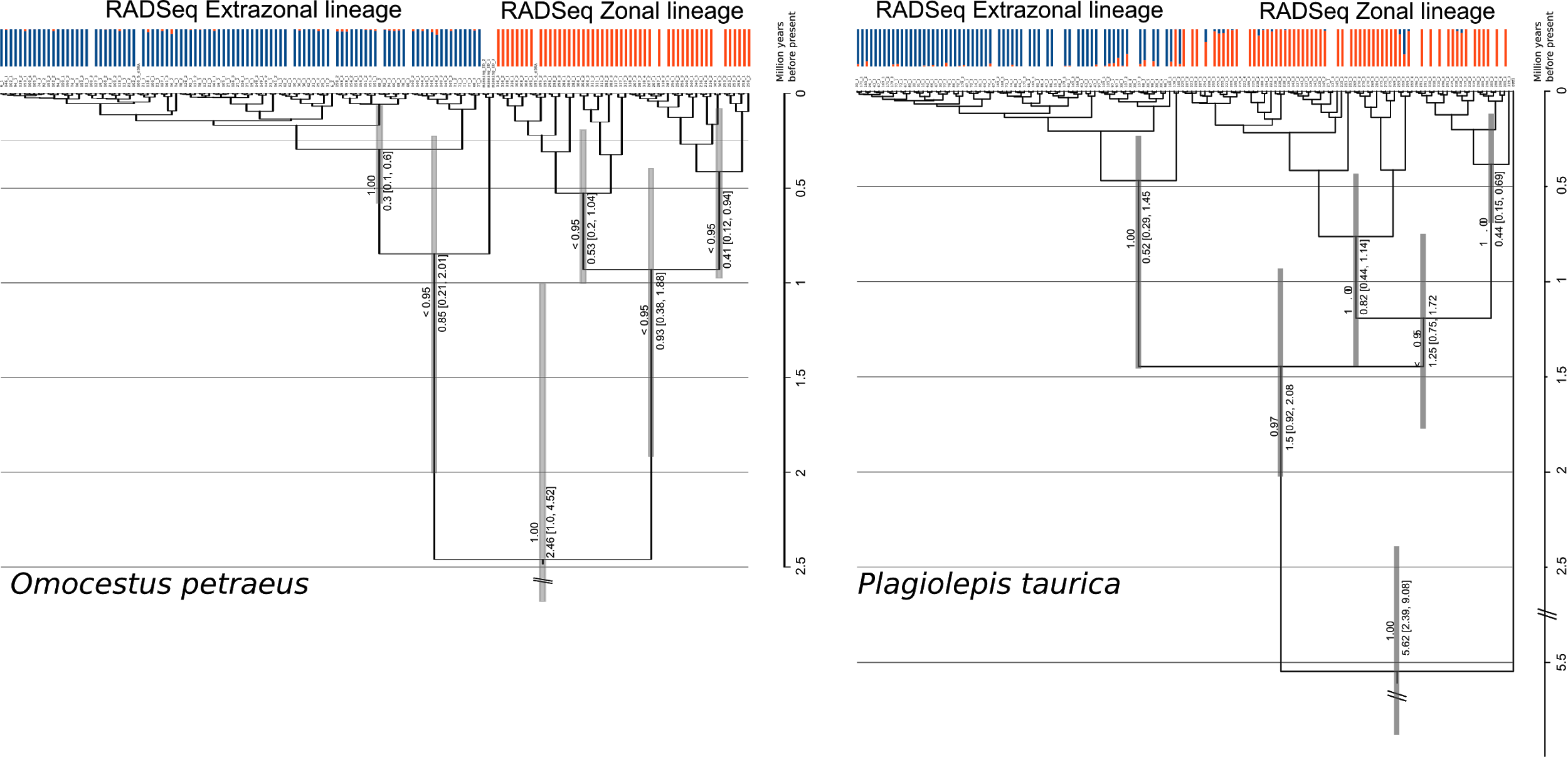


Supplementary Figure 3. Dated Bayesian phylogenies based on parts of the mitochondrial cytochrome oxidase subunit 1 gene. Grey bars depict the 95% highest posterior density intervals. Node support as posterior probability is always given left of the respective bar; median age in million years ago, and in brackets, the corresponding minima and maxima, are given right of the bar (all shown for major splits only). Bars above the trees indicate the affiliation of the respective tip to the corresponding genetic cluster [as obtained via Bayesian clustering based on Restriction Site Associated DNA Sequencing (RADseq) data, Figure 3].


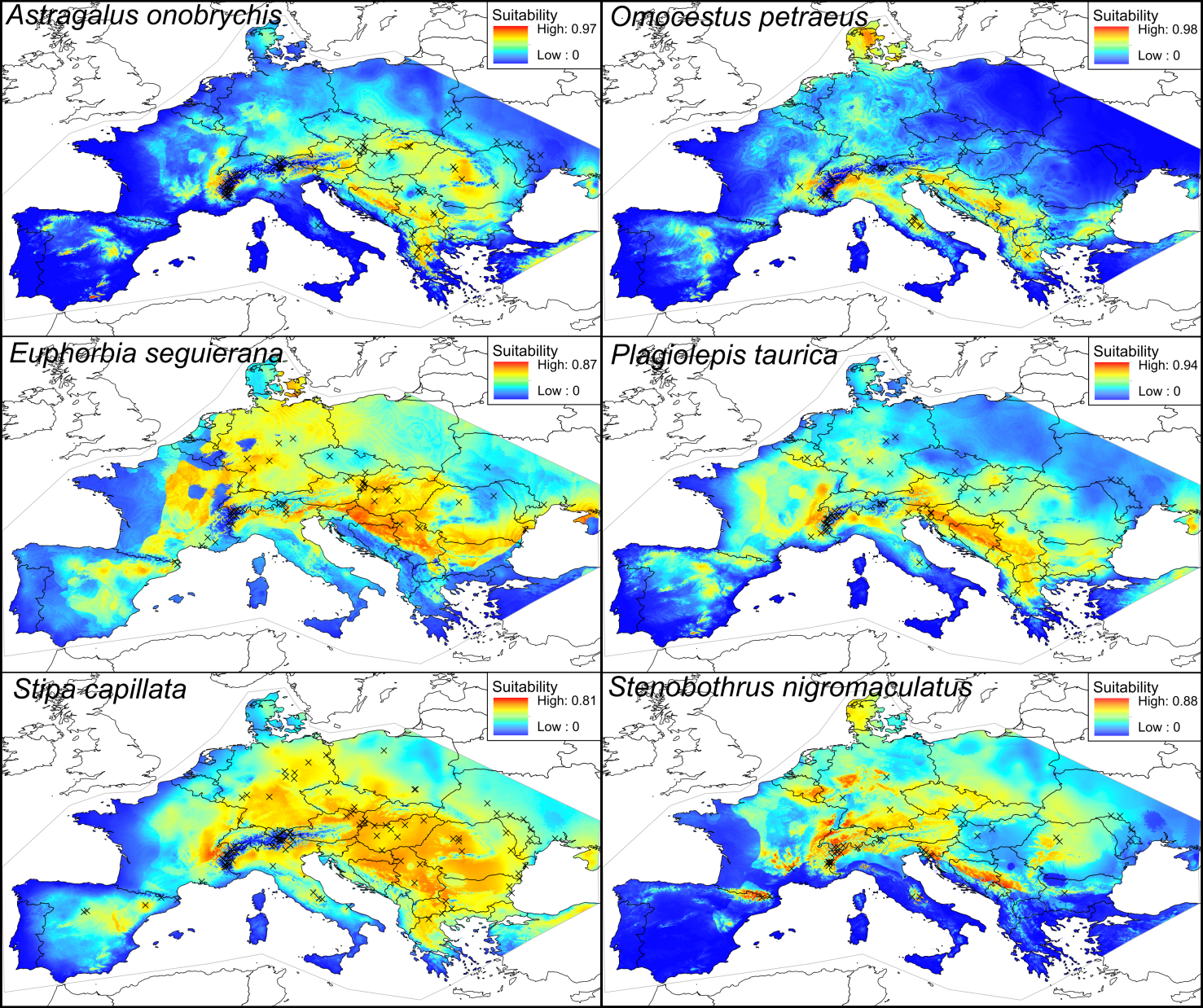


Supplementary Figure 4. Modelled European distribution of the six studied steppe species under current conditions. Source data are provided as a Source Data file.


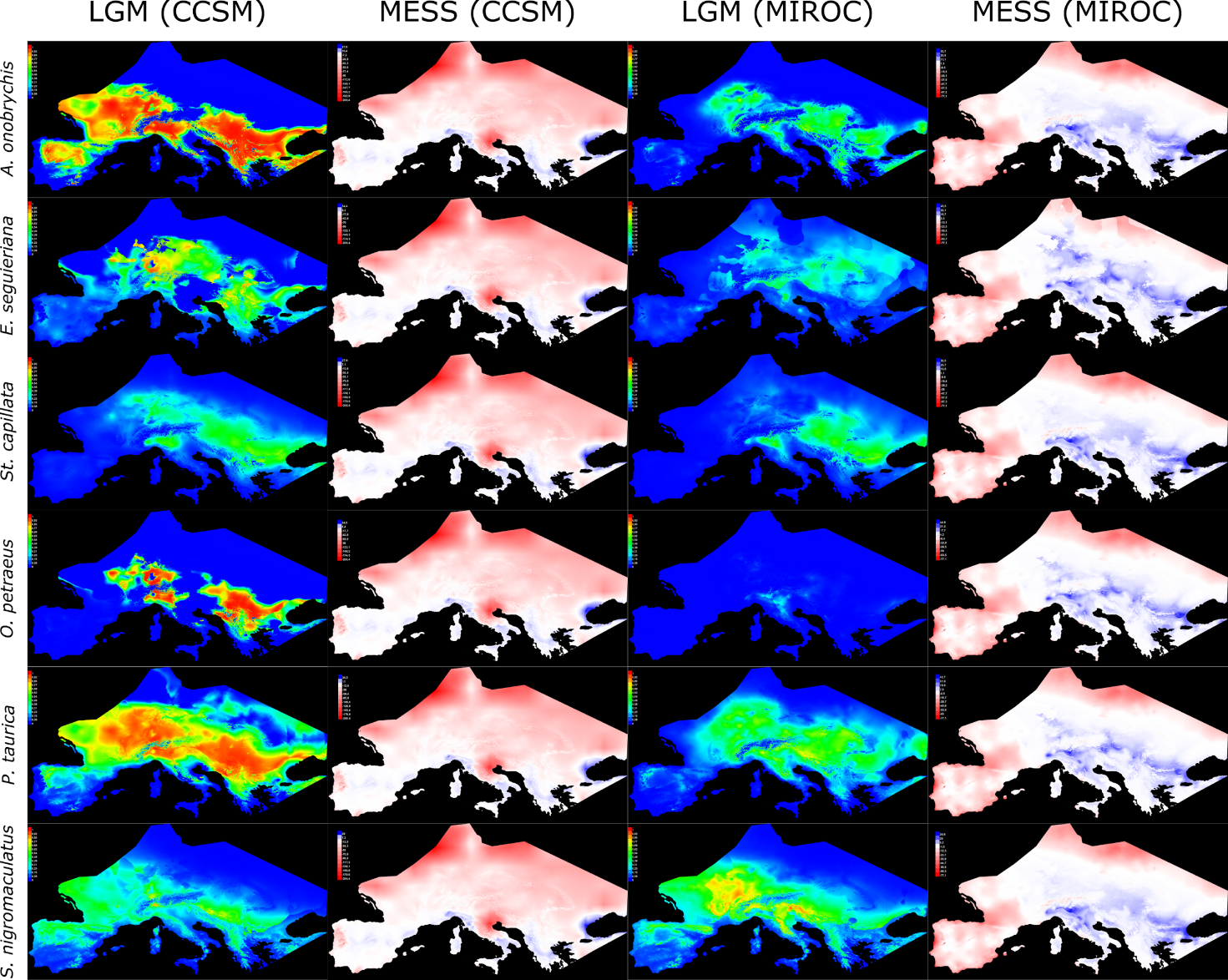


Supplementary Figure 5. Individual niche models projected onto Last Glacial Maximum (LGM). Shown models are based on either bioclimatic conditions from Community Climate System Model (CCSM) or Model for Interdisciplinary Research On Climate (MIROC)^23^⁠. MESS analyses^9^⁠ show no large effect of environmental variables outside the range of the training data upon the model projections. Negative MESS values (red) indicate no-analogue climatic conditions while positive values (blue) indicate analog ones between current and past climatic conditions and the species localities.


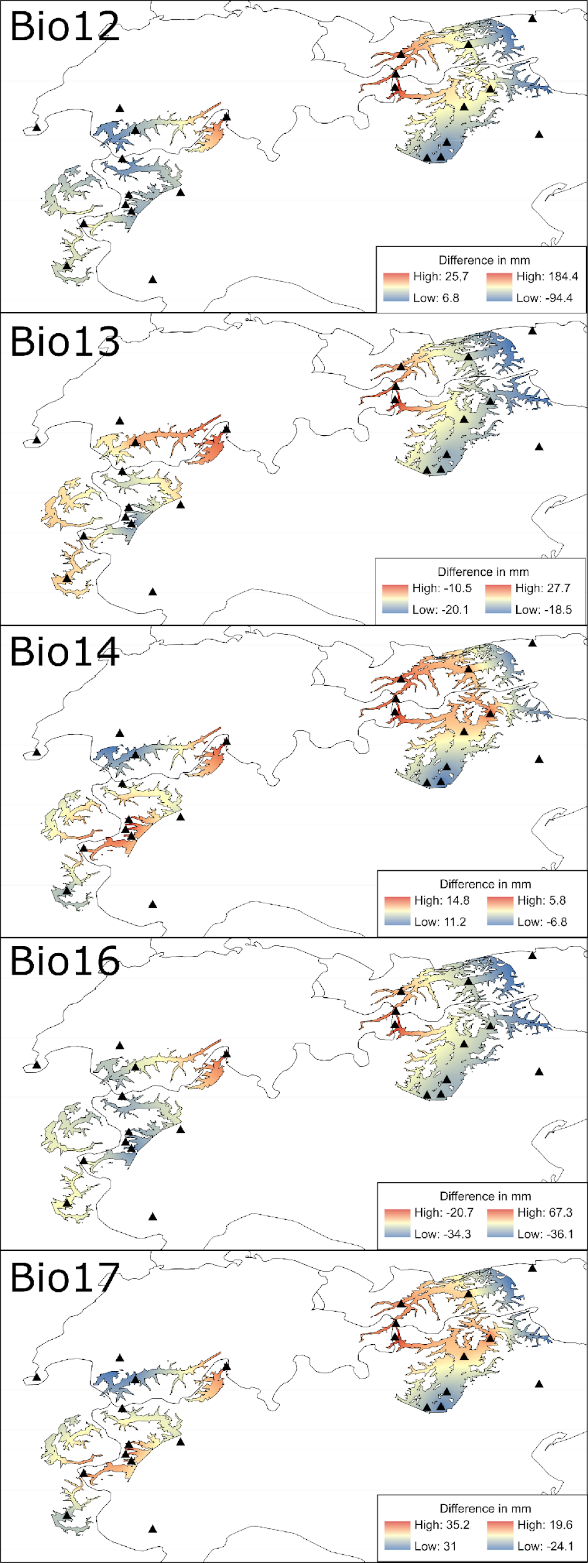


Supplementary Figure 6. Difference surfaces of interpolated climate station data, and values from Worldclim^1^⁠, for five precipitation-related bioclim variables within the limits of selected inner-Alpine dry valleys. In each panel’s legend, the left scale applies to the western, and the right scale to the eastern valleys. Compared to the original bioclim variables, positive values indicate a decrease of precipitation, while positive values indicate an increase of precipitation, after interpolation. Black triangles indicate the locations of the 23 climate stations from which climate data was obtained.

# Supplementary References

1. Hijmans, R. J., Cameron, S. E., Parra, J. L., Jones, P. G. & Jarvis, A. Very high resolution interpolated climate surfaces for global land areas. *Int. J. Climatol.* **25,** 1965–1978 (2005).

2. O’Donnell, M. S. & Ignizio, D. A. Bioclimatic Predictors for Supporting Ecological Applications in the Conterminous United States. U.S. Geological Survey Data Series 691. *US Geol. Surv. Data Ser.* **691,** 10 (2012).

3. Krivoruchko, K. Empirical Bayesian Kriging implemented in ArcGIS Geostatistical Analyst. (2012).

4. Anderson, R. P. & Gonzalez, I. Species-specific tuning increases robustness to sampling bias in models of species distributions: An implementation with Maxent. *Ecol. Modell.* **222,** 2796–2811 (2011).

5. Warren, D. L. & Seifert, S. N. Ecological niche modeling in Maxent: The importance of model complexity and the performance of model selection criteria. *Ecol. Appl.* **21,** 335–342 (2011).

6. Merow, C., Smith, M. J. & Silander, J. A. A practical guide to MaxEnt for modeling species’ distributions: What it does, and why inputs and settings matter. *Ecography (Cop.).* **36,** 1058–1069 (2013).

7. Radosavljevic, A. & Anderson, R. P. Making better Maxent models of species distributions: Complexity, overfitting and evaluation. *J. Biogeogr.* **41,** 629–643 (2014).

8. Muscarella, R. *et al.* ENMeval: An R package for conducting spatially independent evaluations and estimating optimal model complexity for Maxent ecological niche models. *Methods Ecol. Evol.* **5,** 1198–1205 (2014).

9. Elith, J., Kearney, M. & Phillips, S. The art of modelling range-shifting species. *Methods Ecol. Evol.* **1,** 330–342 (2010).

10. John, C., Yeqiao, W. & V., A. P. Assessing current and projected suitable habitats for tree-of-heaven along the Appalachian Trail. *Philos. Trans. R. Soc. B Biol. Sci.* **369,** 20130192 (2014).

11. Brambilla, M., Bergero, V., Bassi, E. & Falco, R. Current and future effectiveness of Natura 2000 network in the central Alps for the conservation of mountain forest owl species in a warming climate. *Eur. J. Wildl. Res.* **61,** 35–44 (2015).

12. McCormack, J. E., Zellmer, A. J. & Knowles, L. L. Does niche divergence accompany allopatric divergence in Aphelocoma jays as predicted under ecological speciation?: Insights from tests with niche models. *Evolution (N. Y).* **64,** 1231–1244 (2010).

13. Johnson, S. E., Delmore, K. E., Brown, K. A., Wyman, T. M. & Louis, E. E. Niche Divergence in a Brown Lemur (Eulemur spp.) Hybrid Zone: Using Ecological Niche Models to Test Models of Stability. *Int. J. Primatol.* **37,** 69–88 (2016).

14. Angelo Canty and B. D. Ripley. boot: Bootstrap R (S-Plus) Functions. (2019).

15. Niklfeld, H. in *Atlas der Donauländer: Karte 171 + Textblatt.* (Österreichisches Ost– und Südosteuropa–Institut, 1974).

16. Meusel, H., Jäger, E. J., Rauschert, S. & Weinert, E. *Vergleichende Chorologie der zentraleuropäischen Flora III*. (Gustav Fischer, 1992).

17. Braun-Blanquet, J. *Die inneralpine Trockenvegetation : von der Provence bis zur Steiermark / Von J. Braun-Blanquet*. (Gustav Fischer, 1961).

18. Ollero, H. S. & van Staalduinen, M. A. in *Eurasian Steppes. Ecological Problems and Livelihoods in a Changing World* (eds. Werger, M. J. A. & van Staalduinen, M. A.) 273–286 (Springer, 2012). doi:10.1007/978-94-007-3886-7_9

19. Catchen, J., Hohenlohe, P. A., Bassham, S., Amores, A. & Cresko, W. A. Stacks: An analysis tool set for population genomics. *Mol. Ecol.* **22,** 3124–3140 (2013).

20. Folmer, O., Black, M., Hoeh, W., Lutz, R. & Vrijenhoek+, R. *DNA primers for amplification of mitochondrial cytochrome c oxidase subunit I from diverse metazoan invertebrates*. *Mol. Mar. Biol. Biotechnol.* **3,** (1994).

21. Lunt, D. H., Zhang, D. X., Szymura, J. M. & Hewitt, G. M. The insect cytochrome oxidase I gene: Evolutionary patterns and conserved primers for phylogenetic studies. *Insect Mol. Biol.* **5,** 153–165 (1996).

22. Pritchard, J. K., Stephens, M. & Donnelly, P. Inference of Population Structure Using Multilocus Genotype Data. *Genetics* **155,** 945 LP-959 (2000).

23. Schmatz, D. R., Luterbacher, J., Zimmermann, N. E. & Pearman, P. B. Gridded climate data from 5 GCMs of the Last Glacial Maximum downscaled to 30 arc s for Europe. *Clim. Past Discuss.* **11,** 2585–2613 (2015).
