# Supplementary material for: Long-term isolation of European steppe outposts boosts the biome’s conservation value
Source: Nat Commun. 2020 Apr 23;11:1968. doi: 10.1038/s41467-020-15620-2 (PMC7181837; doi:10.1038/s41467-020-15620-2)
Supplement: Supplementary file 3 — Description of Additional Supplementary Information [file 41467_2020_15620_MOESM3_ESM.pdf]

Description of Additional Supplementary Files

File Name: Supplementary Data 1

Description: Information on the populations of the studied taxa: locality and collectors, inclusion in Ecological Niche Modelling (ENM), inclusion in Restriction Site Associated DNA sequencing (RADseq) and respective analyses (bayesian Clustering, phylogenetic tree construction), inclusion in mitochondrial DNA sequencing, and NCBI Short Read Archive accession number. Species abbreviations: AO = Astragalus onobrychis, ES = Euphorbia seguieriana, SC = Stipa capillata, OmP = Omocestus petraeus, PT = Plagiolepis taurica, SN = Stenobothrus nigromaculatus.
