# Supplementary material for: Long-term isolation of European steppe outposts boosts the biome’s conservation value
Source: Nat Commun. 2020 Apr 23;11:1968. doi: 10.1038/s41467-020-15620-2 (PMC7181837; doi:10.1038/s41467-020-15620-2)
Supplement: Supplementary file 5 — Reporting Summary [file 41467_2020_15620_MOESM5_ESM.pdf]

nature research

Corresponding author(s): Philipp Kirschner, philipp.kirschner@gmail.com

Last updated by author(s): Feb 24, 2020

Reporting Summary

Nature Research wishes to improve the reproducibility of the work that we publish. This form provides structure for consistency and transparency in reporting. For further information on Nature Research policies, see Authors & Referees and the Editorial Policy Checklist .

Statistics

For all statistical analyses, confirm that the following items are present in the figure legend, table legend, main text, or Methods section.

n/a	Confirmed	
☐	☒	The exact sample size (*n*) for each experimental group/condition, given as a discrete number and unit of measurement
☐	☒	A statement on whether measurements were taken from distinct samples or whether the same sample was measured repeatedly
☐	☒	The statistical test(s) used AND whether they are one- or two-sided<br>*Only common tests should be described solely by name; describe more complex techniques in the Methods section.*
☒	☐	A description of all covariates tested
☒	☐	A description of any assumptions or corrections, such as tests of normality and adjustment for multiple comparisons
☒	☐	A full description of the statistical parameters including central tendency (e.g. means) or other basic estimates (e.g. regression coefficient) AND variation (e.g. standard deviation) or associated estimates of uncertainty (e.g. confidence intervals)
☒	☐	For null hypothesis testing, the test statistic (e.g. *F*, *t*, *r*) with confidence intervals, effect sizes, degrees of freedom and *P* value noted<br>*Give P values as exact values whenever suitable.*
☐	☒	For Bayesian analysis, information on the choice of priors and Markov chain Monte Carlo settings
☒	☐	For hierarchical and complex designs, identification of the appropriate level for tests and full reporting of outcomes
☒	☐	Estimates of effect sizes (e.g. Cohen's *d*, Pearson's *r*), indicating how they were calculated

Our web collection on statistics for biologists contains articles on many of the points above.

Software and code

Policy information about availability of computer code

Data collection	No software was used for data collection.
Data analysis	All tools and software packages that were used for data analysis are given in the method section and the supplementary information, respectively. A quick summary: Bamindexdecoder v. 1.03, Stacks 1.46, Repeatmasker v. 4.0.9, Structure 2.3.4, RaxML v. 8.2.8, Maxent v. 3.3.3.k, PDA 1.0.3, smart model-selection algorithm v. 1.0, Beast v. 1.8, R (packages: ENMeval, phylorare, phrynomics), Passage2 v. 2.0.11.6, Stampy v. 1.0.20, Samtools 1.7, jModelTest v. 2.1.4, Tracer v. 1.5, LogCombiner v. 1.7.5, TreeAnnotator v. 1.7.5, ENMTools v. .1.4.4, fastpcr v. 6.0, ArcGIS v. 10. 4<br><br>Custom R code was used to downsample and plot Phylogenetic Diversity Complemetarity. This Code has been made available on GitHub (https://github.com/philippkirschner/PD_downsampler) as stated in the code availability statement.

For manuscripts utilizing custom algorithms or software that are central to the research but not yet described in published literature, software must be made available to editors/reviewers. We strongly encourage code deposition in a community repository (e.g. GitHub). See the Nature Research guidelines for submitting code & software for further information.

Data

Policy information about availability of data

All manuscripts must include a data availability statement. This statement should provide the following information, where applicable:
- Accession codes, unique identifiers, or web links for publicly available datasets
- A list of figures that have associated raw data
- A description of any restrictions on data availability

Demultiplexed RADseq sequencing data and mitochondrial DNA sequences are available from the NCBI GenBank Short Read Archive, and NCBI Nucleotide Database, respectively (all accession numbers in Supplementary Table 1). Other data and results are included in the Supplementary Material.
The climate data that has been used for layer interpolation is publicly available upon request from Zentralanstalt für Meteorologie und Geodynamik (Austria, ZAMG;

www.zamg.ac.at), Federal Office of Meteorology and Climatology (Switzerland, MeteoSwiss; http://www.meteosuisse.admin.ch), and Météo-France (France, http://www.meteofrance.com). The values as used for interpolation are listed in Supplementary Table 8.

Field-specific reporting

Please select the one below that is the best fit for your research. If you are not sure, read the appropriate sections before making your selection.

☐ Life sciences ☐ Behavioural & social sciences ☒ Ecological, evolutionary & environmental sciences

For a reference copy of the document with all sections, see nature.com/documents/nr-reporting-summary-flat.pdf

Ecological, evolutionary & environmental sciences study design

All studies must disclose on these points even when the disclosure is negative.

Study description	We apply a complementary approach, combining genomic data (RadSeq), phylogenetic (RaxML) and population genetic (STRUCTURE) inference, as well as ecological modelling across six steppe species representing different phyla, families, and genera sampled across the Eurasian steppe biome (total of 1036 individuals from total of 380 populations) in order to prioritize among different parts of the Eurasian steppes for their conservation
Research sample	The selected species (Astragalus onobrychis, Euphorbia seguieriana, Stipa capillata, Omocestus petraeus, Plagiolepis taurica, Stenobothrus nigromaculatus) are typical inhabitants of dry-continental Eurasian steppes, some of which have wider distribution areas spanning most of Eurasia (e.g., Stipa capillata), while others are geographically more restricted. The study focuses on vascular plants, grasshoppers and ants, i.e., on organism groups representing a range of different life history traits. As a population, we understand the set of individuals inhabiting the locality specified in the Supplementary Table 1 uniquely assigned by the locality ID. Grasshoppers were collected as adults, as larval and subadult stages do not allow unambiguous identification on species level. Usually both sexes were collected, except when permits did not allow collection of females. In case of the ant P. taurica, worker ants (females) were collected.
Sampling strategy	All species were sampled across their distribution ranges, while extrazonal occurrences were more densely sampled than zonal ones. Three randomly taken individuals were sampled per population. As sequencing ressources were a limiting factor, we chose a sample size that allowed us to still detect genetic variability within a population, and at the same time, allowed us to sequence as many populations as possible from the steppe biome.
Data collection	All collectors are listed in Supplementary Table 1.
Timing and spatial scale	Samples were collected mostly between 2014 and 2016 with earlier sampling for trial analyses (21.6.2001 - 23.10.2015). The sampling time has no influence on the analytical approaches we applied. The area of sampling reflects the vast steppe biome (i.e. Westernmost sampling site: Northeastern Iberian Peninsula, Spain; Easternmost sampling site: steppic habitats in the foothills of Tian Shan mountains Kyrgyzstan).
Data exclusions	No data were excluded from the analyses
Reproducibility	From each species-specific genetic (RadSeq) datasets 10% of the samples were replicated. After sucessful evaluation of the reproducibility of the results the replicates were removed from the final datasets.
Randomization	As our study is based on field-collected samples, no randomisation was necessary.
Blinding	Blinding was not necessary for this study

Did the study involve field work? ☒ Yes ☐ No

Field work, collection and transport

Field conditions	Field conditions were not considered to be relevant for the conducted sampling. Generally days without precipitation were favoured to collect insects though.
Location	All sampled localities and their coordinates are given in Supplementary Table 1
Access and import/export	Acquired permits are acknowledged in the main manuscript. (utonomous Province Bozen, Italy (No. 333338), the Autonomous Region Vallé d'Aosta, Italy (14191/Rr), the Austrian federal state Burgenland (5-N-A1007/586-2014), the Austrian federal state Lower Austria (RU5-BE-1049/001-2014), the German federal state Rheinland-Pfalz (Nord: 425-104.141.1402, South: 42/553-251), the German federal state Thuringia, and the canton Grisons, Switzerland (AV-2014-210)).
Disturbance	Plants: From all plant individuals sampled 1-2 fresh leaves were collected from 5 mature plants per population. For herbarium specimens one ramet (typically: flowering shoot) per population was collected with the exception of populations that were too small (< 20 individuals). Ants: From Plagiolepis ant nests, a maximum 20-30 workers were removed for analysis, nests were never completely extracted or destroyed, gynes were generally not sampled. A maximum of 5 nests per site was sampled. Grasshoppers: From each sampled site, a maximum of 5 specimens was sampled. In few instances when population sizes were

small, sampling was conducted after the reproductive season was completed (i.e. early October), and in these cases, only males were sampled

Reporting for specific materials, systems and methods

We require information from authors about some types of materials, experimental systems and methods used in many studies. Here, indicate whether each material, system or method listed is relevant to your study. If you are not sure if a list item applies to your research, read the appropriate section before selecting a response.

Materials & experimental systems

n/a	Involved in the study
☒	☐ Antibodies
☒	☐ Eukaryotic cell lines
☒	☐ Palaeontology
☒	☐ Animals and other organisms
☒	☐ Human research participants
☒	☐ Clinical data

Methods

n/a	Involved in the study
☒	☐ ChIP-seq
☒	☐ Flow cytometry
☒	☐ MRI-based neuroimaging
